# MEDICI: Mining Essentiality Data to Identify Critical Interactions for Cancer Drug Target Discovery and Development

**DOI:** 10.1371/journal.pone.0170339

**Published:** 2017-01-24

**Authors:** Sahar Harati, Lee A. D. Cooper, Josue D. Moran, Felipe O. Giuste, Yuhong Du, Andrei A. Ivanov, Margaret A. Johns, Fadlo R. Khuri, Haian Fu, Carlos S. Moreno

**Affiliations:** 1 Department of Biomedical Informatics, Emory University, Atlanta, Georgia, United States of America; 2 Graduate Program in Biomedical Informatics, Emory University, Atlanta, Georgia, United States of America; 3 Winship Cancer Institute, Emory University, Atlanta, Georgia, United States of America; 4 Department of Biomedical Engineering, Emory University, Atlanta, Georgia, United States of America; 5 Graduate Program in Cancer Biology, Emory University, Atlanta, Georgia, United States of America; 6 Department of Pathology and Laboratory Medicine, Emory University, Atlanta, Georgia, United States of America; 7 Medical Scientist Training Program, Emory University, Atlanta, Georgia, United States of America; 8 Department of Pharmacology, Emory University, Atlanta, Georgia, United States of America; 9 Department of Hematology & Medical Oncology, Emory University, Atlanta, Georgia, United States of America; Flinders University, AUSTRALIA

## Abstract

Protein-protein interactions (PPIs) mediate the transmission and regulation of oncogenic signals that are essential to cellular proliferation and survival, and thus represent potential targets for anti-cancer therapeutic discovery. Despite their significance, there is no method to experimentally disrupt and interrogate the essentiality of individual endogenous PPIs. The ability to computationally predict or infer *PPI essentiality* would help prioritize PPIs for drug discovery and help advance understanding of cancer biology. Here we introduce a computational method (MEDICI) to predict *PPI essentiality* by combining gene knockdown studies with network models of protein interaction pathways in an analytic framework. Our method uses network topology to model how gene silencing can disrupt PPIs, relating the unknown essentialities of individual PPIs to experimentally observed protein essentialities. This model is then deconvolved to recover the unknown essentialities of individual PPIs. We demonstrate the validity of our approach via prediction of sensitivities to compounds based on PPI essentiality and differences in essentiality based on genetic mutations. We further show that lung cancer patients have improved overall survival when specific PPIs are no longer present, suggesting that these PPIs may be potentially new targets for therapeutic development. Software is freely available at https://github.com/cooperlab/MEDICI. Datasets are available at https://ctd2.nci.nih.gov/dataPortal.

## Introduction

Advances in high-throughput screening technology have enabled broad investigations of genome-wide gene/protein essentiality in cancer. High-throughput single-gene shRNA/siRNA silencing [1–4] and CRISPR-Cas9 inactivation [5] are well-established experimental approaches to study protein essentiality in genome-wide screens. Observing the proliferative effects of silencing each gene/node in a PPI network can provide insights into tumor biology and help identify promising therapeutic targets, especially when combined with genomic characterizations. Whole-genome siRNA screens have been combined with genomic profiles and drug screens in lung adenocarcinoma to identify context-specific drug sensitivities and their genetic biomarkers [6]. Project Achilles currently provides a pooled shRNA screening database with more than 11,000 genes in 216 cell lines [7]. Systematic analyses of these data have been able to identify specific gene vulnerabilities within genetic contexts in several studies [7–11].

The PPI interface has become increasingly recognized as a tractable target for small molecules therapeutics, as evidenced by recent clinical development of p53/MDM2 and BET bromodomain small molecule inhibitors [2, 12, 13]. Despite the therapeutic potential of protein-protein interactions (PPIs) as drug targets [14], specific analysis of protein-interaction essentiality or the essentiality of *interactions* in biological networks (‘edgetics’) is in its infancy [15, 16]. Current technologies focus on silencing of single genes in large-scale shRNA screens; however, shRNA silencing of a single gene effectively disrupts multiple PPIs and masks the contributions of individual PPIs to the overall protein essentiality. High-throughput technology for interrupting specific PPIs on a whole-interactome scale does not exist, and methods for experimentally measuring the essentiality of individual endogenous PPIs at the genome scale will likely remain an unsolved problem for the foreseeable future. While large-scale PPI screens have measured the effects of disease mutations on specific PPIs [15, 16], they do not provide data on the essentiality of endogenous interactions for the survival of a cell. Thus, we were motivated to develop a computational approach to estimate the essentiality of PPIs by integrating PPI network topology with whole-genome shRNA screens. By measuring the essentiality of every gene (node) in a network, and understanding how proteins are connected through protein interactions (edges), we aim to estimate the essentiality of individual PPIs that are silenced in aggregate as a gene is knocked down by shRNA.

The integration of functional screens with PPI networks has been previously explored with an emphasis on mitigating screening noise to improve the robustness of functional measurements. PPI networks have been integrated with RNAi screens using a diffusion kernel-based method [17] to successfully reduce false-positive and false-negative results in *Drosophila* screens. The IMPACT method used protein interactions as a method for reducing off-target effects and improving the biological interpretation of screened phenotypes [18]. In addition, KEGG networks have been integrated with siRNA screens to refine the insulin-signaling network using a network seeding/pruning approach [18]. A shortest path approach for analysis of PPI networks has been developed and applied to pancreatic cancer [19]. Furthermore, the NEST approach improves on CRISPR data for analysis of gene or node essentiality [20]. However, to our knowledge, no available method leverages genome-scale functional screening resources to compute the importance of individual PPIs within biological networks. Here we describe MEDICI (Mining Essentiality Data to Identify Critical Interactions), a new computational approach that leverages high-throughput gene knockdown data and protein interaction network topology to rank PPIs based on their criticality for the survival and proliferation of cancer cells. This approach does not predict new PPIs, but rather ranks the relative importance of known PPIs within a cancer cell for cell survival.

## Methods

We obtained shRNA knockdown measurements from Project Achilles [7] v2.4.3 (http://www.broadinstitute.org/achilles). The.gct file containing multiple shRNAs for each gene (Achilles_QC_v2.4.3.rnai.Gs.gct) was analyzed to produce gene-level measurements. We used the value of the second most lethal shRNA for each gene to mitigate spurious off-target effects as previously described [7]. Moreover, the gene solutions for the shRNAs in this dataset have also been corrected for off-target effects using ATARiS software [21]. Cell line metadata was obtained from both Achilles and the Cancer Cell Line Encyclopedia[22] (CCLE, http://www.broadinstitute.org/ccle), including histology and organ site, mutations, copy number variation and sensitivities to compounds in the CCLE library.

The superpathway was constructed by combining curated pathway models from the NCI Pathway Interaction Database [23] (PID, http://pid.nci.nih.gov/) which can be accessed via MSigDB (http://software.broadinstitute.org/gsea/msigdb) [24] with protein-interaction screening results obtained via TR-FRET [25]. The gene symbols for the PID pathway were first mapped to the official HUGO symbols to resolve ambiguity and aid in integration with the Achilles dataset. Pathway entities that could not be mapped to HUGO were excised from the pathway model. All 196 PID pathways were aggregated to form a superpathway with 1548 nodes and 7906 interactions. We added 208 novel interactions identified in PPI screening [25] to this superpathway and annotated them for future reference. We used mutations and copy number alterations from CCLE datasets [22] to remove lost nodes and build 206 cell line context specific networks. Achilles shRNA data was then integrated with each network to produce a gene-essentiality layered superpathway for each cell line. Pathway nodes that did not have corresponding shRNA measurements were excised from the network model. The final set of 7906 PPI essentialities across 206 cell lines is available in S1 Table. All datasets used in the analysis are available for download at the Cancer Target Discovery and Development (CTD2) Data Portal (https://ctd2.nci.nih.gov/dataPortal/). We have also conveniently packaged the data necessary for reproducing the analysis on Synapse. We are currently developing a PPI Portal website where PPI essentiality data, as well as data from our OncoPPi screening study [25], can be mined and viewed (A. Ivanov, B. Revennaugh, et al, manuscript in preparation). This website will allow users to export data for analysis in excel or in Cytoscape, or to capture network images as png files.

### Modeling PPI network topologies

Biological networks consist of proteins (as nodes) and interactions (as edges) between them. For this research, we analyze the interactions between proteins with consideration of all other interactions and proteins in the network. To begin, we first consider a PPI between two proteins. These two proteins are typically also involved in interactions with other nodes. If a PPI is essential, then it’s more likely that other interactions of those two proteins are essential. Furthermore, one would expect the interactions happening in proximity of that interaction are also essential. In other words, there is a locality of essentiality for PPIs.

**Fig 1:**
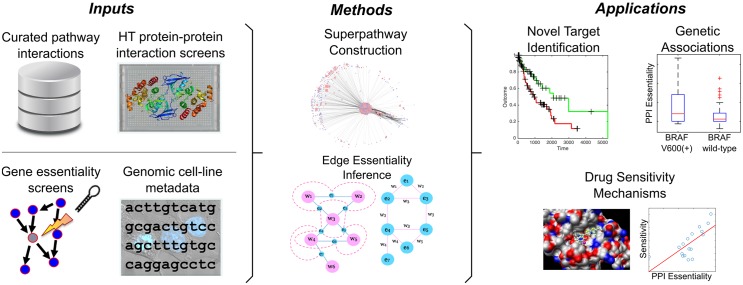
Details of the computational framework of MEDICI. Curated pathway descriptions are integrated with novel interactions discovered by PPI screening to generate an interaction superpathway. Gene essentiality measurements are layered onto the nodes of the superpathway, and the network topology is transformed to the dual graph where the genes become network edges and the gene-interactions become network nodes. Gene essentialities are then diffused over their interactions to infer interaction essentiality weights.

To proceed we make the locality of PPIs more formal by inverting the pathway network and representing each of the PPIs as nodes, and each of the proteins as edges. Two PPIs are connected in the network if they share a protein, i.e., each edge in the new graph corresponds to a protein. We thus generate a new graph encoding the proximity for PPIs (Fig 1). Because we expect that PPIs that are connected with more essential proteins would have higher correlation in essentiality, we choose the edge weights in the dual network to be proportional to the essentiality of the corresponding protein derived from the shRNA screening data.

Once we have constructed our model with the dual graph, we infer the essentiality of PPIs in an unsupervised manner. Following the well-known PageRank algorithm [26], in order to promote the locality of essentiality, we employ an iterative update of essentialities. At each step, the essentiality of each node (or PPI) is updated to be the average of the localities of its neighbors. This is consistent with our intuition of the locality of the essentiality score. If an interaction is essential, it is likely that the interactions nearby are also vital. Furthermore, a less essential interaction is usually close to less important ones. In other words nearby interactions are correlated and the amount of this correlation is proportional to the essentiality of nodes in between and the structure of the network. More interestingly, these updates are structurally motivated, i.e., the scores derived reflect the structural importance of the PPIs in the whole interaction network.

### Calculate PPI essentialities and ranking

A dual network is constructed from the given network, i.e., the network is transformed so that each PPI/edge is represented by a node. Two nodes in the dual graph are connected if and only if their corresponding edges (in the original graph) have a shared end-point. Inspired by the locality of the essentiality, an iterative method is proposed in which the essentiality of a node in the dual graph is averaged over the essentiality of its direct neighbors. Specifically, we define the essentiality of node *i* (in the dual graph) at *t*th step by $e_{i}^{\left(t\right)}$. Also let $e_{i}^{\left(0\right)}$ be a random initialization between 0 and 1. At step (*t* + 1) the score of each node is the weighted average of the score of its neighbors smoothed by its value in previous step with 0 < *α* ≤ 1.0. Intuitively, the significance of each node propagates to its neighbors.

For, example in the network given by Fig 1 the essentiality for node 1 and 2 of the dual graph (edges *e*_1_ and *e*_2_ in the original network) are updated as follows:

$$e_{1}^{\left(t+1\right)}=\left(1-\alpha \right)\;e_{1}^{\left(t\right)}+\alpha \left(\frac{p_{1}\;e_{2}^{\left(t\right)}+p_{2}\;e_{3}^{\left(t\right)}}{p_{1}+p_{2}}\right)$$

$$e_{2}^{\left(t+1\right)}=\left(1-\alpha \right)\;e_{2}^{\left(t\right)}+\alpha \left(\frac{p_{1}\;e_{1}^{\left(t\right)}+p_{3}\;e_{3}^{\left(t\right)}+p_{3}\;e_{4}^{\left(t\right)}}{p_{1}+p_{2}+p_{3}}\right)$$

The weight of each edge is influenced by the essentiality of the protein. i.e, we have an exponential kernel *exp*(*w* * *p*_*i*_), where *p*_*i*_ is the essentiality of protein *i* and *w* is the coefficient. By setting different *w* we can incorporate our prior belief on the importance of the essentiality of the protein in the essentiality of the edges.

$$e_{1}^{\left(t+1\right)}=\left(1-\alpha \right)\;e_{1}^{\left(t\right)}+\alpha \left(\frac{exp(w*p_{1})\;e_{2}^{\left(t\right)}+exp\left(w*p_{2}\right)\;e_{3}^{\left(t\right)}}{exp(w*p_{1})+exp\left(w*p_{2}\right)}\right)$$

$$e_{2}^{\left(t+1\right)}=\left(1-\alpha \right)\;e_{2}^{\left(t\right)}+\alpha \left(\frac{exp\left(w*p_{1}\right)\;e_{1}^{\left(t\right)}+exp\left(w*p_{3}\right)\;e_{3}^{\left(t\right)}+exp\left(w*p_{3}\right)\;e_{4}^{\left(t\right)}}{exp(w*p_{1})+exp(w*p_{2})+exp\left(w*p_{3}\right)}\right)$$

After sufficiently large number of steps the scores converge and the final score is independent of the initialization. It can be easily seen that the stationary values (when the essentiality computation converges) can be simply computed by Eigenvalue decomposition of a variant of the adjacency matrix of the dual graph.

### Smoothing

The above updates are subject to overfitting and are sensitive to noise. Consequently, at each step the new essentiality value may change dramatically because of the changes in the neighboring PPIs. To control this undesirable behavior, we introduce a smoothing step to the model in order to regularize the solution. At each step, the new value is a weighted sum of the previous value and the value inspired by the neighbors. The smoothing parameter, 0 < *α* ≤ 1, controls this tradeoff between the locality and the regularization. The new value is (1 - *α*) times the previous value plus *α* times the values computed from the neighbors.

We analyzed *α* = 0.9, 0.5, 0.1 and we observed that the smoothing parameter doesn’t have a significant effect on the ranking of essentialities. It only changes the values but won't affect the ordering. A lower *α* will damp out the essentialities and make them close to each other. For example *α* = 0 will result in a uniform essentialities (all equal). The low values of *α* make the update rule reluctant to change the essentialities in iterations and the values change smoothly hence, the final values will be close to each other. On the other hand, as we increase *α* the effect of neighbors will increase, the changes have higher magnitudes, and the difference is more apparent. The smoothing will make the computed values numerically more stable. The eigenvector computation is pruned to degenerate results when the smallest eigenvalue is close to 0. The smoothing will increase the lowest eigenvalue and therefore the convergence computation will not be affected by degenerate cases. Also, the smoothing can be seen as a denoising approach. If the data is noisy the smoothing will remove the fine-tuning due to the noise. Therefore, variations due to the noise will not affect the model.

We determined that a value of *α* = 0.5 was optimal for the analyses in this study. A second parameter *w* represents how much the essentiality of proteins affects the essentiality of interactions in between. In other words, larger values of *w* show that an interaction is essential if the corresponding proteins are essential, and smaller values of *w* show that no matter how essential the corresponding proteins are the interaction is important, e.g., because of its centrality role in the PPI network. Examining different values we set *w* to 0.5. See S1 Methods, where we demonstrate that this iterative update is guaranteed to converge and has a closed form answer. Furthermore the final value is independent of the initialization. The final essentiality score ranges from 0 (not essential) to 1 (completely essential to cell survival). All MEDICI software is available for public download at https://github.com/cooperlab/MEDICI.

### Data mining and statistical tests

We applied MEDICI to shRNA gene-essentiality profiles from 206 cell lines in the Achilles database [8]. A context specific PPI network was created for each cell line by using mutational and CNV events to re-wire the topology of a prior-knowledge superpathway [23] containing 2,186 proteins and 11,488 PPIs. This superpathway was a subset of the NCI Pathway Interaction Database[23] that is now hosted by the public network data exchange [27] (http://www.ndexbio.org/). Gene essentiality measurements were integrated with the re-wired networks, and then deconvolved by constructing a dual-graph to predict unknown PPI essentialities from the measured gene essentialities and the PPI network topology (Fig 1). To evaluate these PPIs in patient samples, we used mutational, CNV and mRNA expression profiles to infer which PPIs are absent in each patient. A Cox proportional hazards analysis was then used to measure the prognostic significance of these groups. PPIs from lung adenocarcinoma derived lines were ranked to identify the most essential PPIs, KS statistics were computed for ranks across these lines, and a positive KS statistic > 0.5 was used as a threshold for significant PPIs in LUAD cell lines.

## Results

### PPI essentiality rankings

We used the context-specific networks to compute the essentialities of 7906 PPIs in 206 cell lines from the project Achilles dataset and have reported these in S1 Table. Context specific networks were based on a superpathway that was constructed by combining 196 curated pathway models from the NCI Pathway Interaction Database with 208 novel interactions identified in PPI screening [25] and mutations and copy number alterations from CCLE datasets [22]. Examination of PPIs with the highest average essentialities determined that the top 20 were dominated by interactions of CTNNB1 and AKT1. Ten of the top 20 PPIs involved CTNNB1, while 8 of 20 involved AKT1. The other two top 20 PPIs were SOS1-SRC and MYC-SMAD4. Highly essential interactions of CTNNB1 in descending order were those with SRC, RAC1, JUN, PTPN11, HDAC1, EGFR, RHOA, CTNNA1, CDC42, and AR. The top interactions of AKT1 were with SRC, RAC1, SOS1, PIK3CA, PTPN11, PIK3R1, JAK1, and MTOR. SRC was a component of 3 of the top 20 PPIs, and the top two PPIs across all cell lines were CTNNB1-SRC and SRC-AKT1, suggesting that the SRC-AKT1-CTNNB1 pathway is of potentially general interest for targeting PPIs. Previous studies have shown synergistic effects using combinations of SRC and AKT inhibitors [28] in small cell lung cancers.

### Clustering analysis

Data were filtered to remove cell lines with large numbers of missing nodes (> 20% missing due to gene mutation or deletion) and PPIs with uniform essentiality across cell lines, resulting in a final matrix of 165 cell lines by 5798 PPIs for PPI essentiality clustering analysis. Unsupervised hierarchical clustering revealed strong clusters of some subsets of PPIs (S1 Fig). In general, the PPIs within these clusters shared a single dominant oncogene (e.g. AKT1 or CTNNB1) or tumor suppressor (e.g. TP53) as one of the interacting partners.

**Fig 2:**
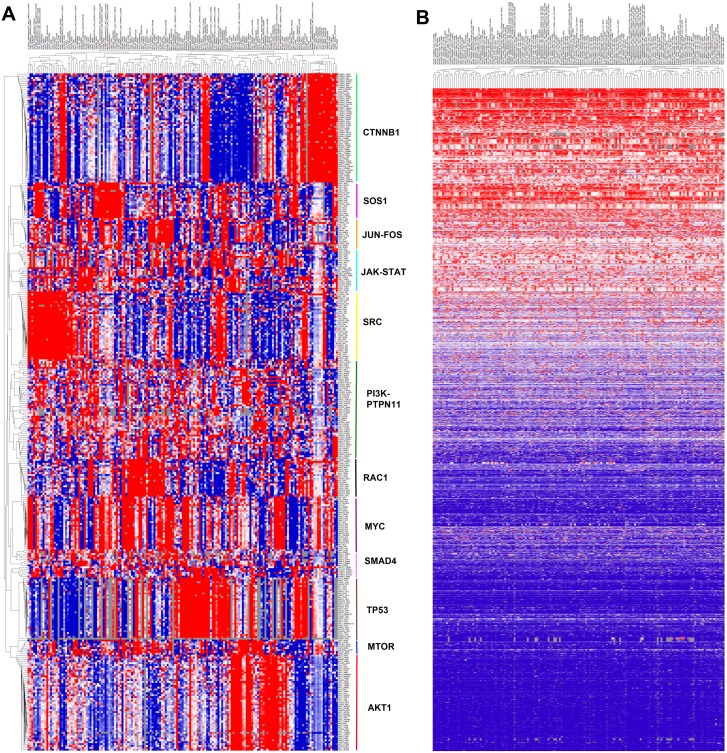
Clustering of most essential PPIs in the superpathway. (A) Unsupervised Hierarchical Clustering of the 360 most essential PPIs across the 165 cell lines identifies 12 major clusters. The 360 PPIs with an average essentiality score > 0.5 were used to cluster 165 cell lines used in the Achilles shRNA screening study using Cluster and Java Treeview software [29]. PPI essentiality data was median centered and clustered by average correlation. Red indicates higher essentiality and blue indicates lower essentiality. Major hubs for each cluster are indicated on the right. (B) Clustering of 5798 PPI-MPER values across 165 cell lines. Red indicates PPI essentiality is greater than the max protein essentiality, and blue indicates the PPI essentiality is less than the max protein essentiality.

To delve further into those PPIs with therapeutic potential, we analyzed the subset of the 360 most essential PPIs with an average essentiality > 0.5 across all cell lines. An essentiality score of 0 is a non-essential PPI, whereas a score of 1 is a completely essential PPI for cell survival. Clustering of these 360 PPIs across the 165 cell lines identified 12 major PPI clusters. Each of these clusters was strongly associated with either a single protein or a single pathway (Fig 2A). Of particular interest, the PPIs among the top 20 most essential often those linked proteins that dominated particular PPI clusters such as AKT1-SRC, AKT1-MTOR, and CTNNB1-SRC. Interestingly, the cell lines did not cluster primarily by tissue of origin, with the exception of colon-derived cell lines that were driven by the large CTNNB1 cluster. Instead, cell line clustering was likely driven by shared activation of oncogenic pathways.

In contrast, when we clustered the 165 cell lines by protein nodes using the gene-centric Achilles shRNA sensitivity data across 5711 genes, the cell lines clustered primarily by tissue of origin (S2 Fig). A few strong clusters were apparent in the unsupervised clustering of Achilles shRNA data, which included sets of transcription factors, ribosomal proteins, or genes of unknown function. To compare the supervised clustering of edges from Fig 2 using shRNA node data, we extracted the 343 proteins with the top average shRNA sensitivity across all cell lines (<-0.05), and performed identical hierarchical clustering (S3 Fig). Cell lines continued to be clustered by tissue of origin, and there were few distinct clusters, with the exception of one small cluster containing MHC class I receptors.

To compare protein and PPI essentiality, we ranked each interaction by its PPI essentiality, and then compared this to the maximum protein essentiality of the interaction source and target. Interactions with a high PPI essentiality but a low source/target essentiality are especially interesting, since these represent vulnerabilities that will not be revealed in single-gene knockdowns. We further computed the ratios of the PPI essentialities to the maximum essentiality of either interacting protein and performed hierarchical clustering of the PPI/maxProteinEssentiality ratio (PPI-MPER) data (Fig 2B). We observed that 2294 (29%) PPIs had a PPI-MPER that was > 1 in over half of the 165 cell lines (S3 Table). Of the 7814 PPIs examined, 6503 (83%) had a PPI-MPER > 1 in at least one cell line. Importantly, 2562 (33%) PPIs had a median PPI-MPER < 0.5, indicating that the max Protein essentiality was 2-fold or more higher than the PPI essentiality in at least half of the cell lines. These data suggest that the MEDICI software is differentiating crucial PPIs from unimportant PPIs, even when they occur between essential proteins. Among the PPIs with consistently high PPI-MPER values were CDK2_RB1 [30], PIK3R1_YWHAZ [31], JAK3_MAPK14, CREBBP_CTNNB1 [32], and HIF1A_SMAD4 [33]. Some of these interactions are well documented as key interactions in critical signal transduction pathways, while others less so, suggesting that they may warrant further investigation.

### Crosstalk of signal transduction pathways

Other PPIs such as YAP1_CTNNB1 [34], AR_CTNNB1 [35, 36], and SMAD7_CTNNB1 [37] were much more essential than either protein (S4 Fig), but in only a subset of cell lines. For example, the AR_CTNNB1 PPI essentiality rank percentile was very high in a number of cell lines including COLO704 ovarian (99%ile), and BT20 (98%ile) and MDA-MB-453 (97%ile) triple-negative breast cancer cell lines. Both Wnt [38] and androgen signaling [39] have been shown to be important in subsets of ovarian and triple-negative breast cancers [40–42]. Another example of signaling crosstalk identified by this analysis is that of YAP1_CTNNB1 interactions in L363 myeloma cells (94%ile), CADO-ES1 Ewing’s sarcoma cells (96%ile), and A204 rhabdomyosarcoma cells (96%ile). Wnt and Hippo signaling are important in myelomas, bone, and soft tissue cancers [43–47]. We also observed potential crosstalk of TGFβ and Wnt signals in colorectal RKO cells (95%ile), KP4 pancreatic cells (97%ile), and 22RV1 prostate cancer cells (99%ile). SMAD7 has been implicated in colon cancer development [48, 49], and in interactions with β-catenin in pancreatic cancer [50] and prostate cancer [37].

### Drug sensitivity

**Fig 3:**
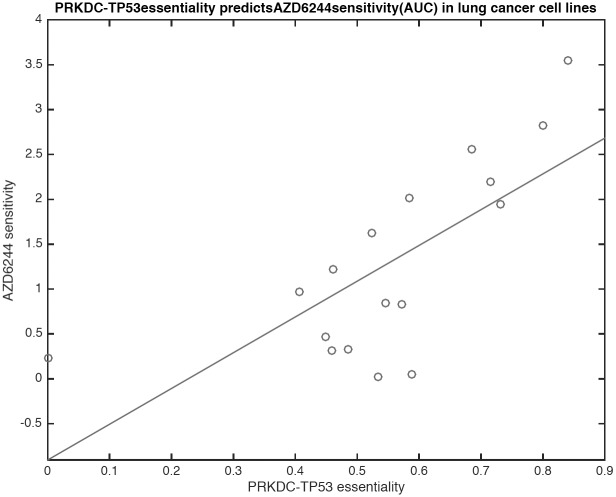
Correlating interaction essentialities with drug sensitivity measures provides insights into mechanisms of action. We correlated drug sensitivity measures from CCLE with interaction essentiality scores to identify critical interactions that predict therapeutic sensitivity. Sensitivity to the MAPK inhibitor AZD6244 is highly correlated with PRKDC-TP53 interaction essentiality, which is consistent with the well established role of p38-MAPK in cell cycle arrest in response to DNA damage [51–53].

The lack of even low-throughput technology to produce PPI essentiality measurements makes validation of targets difficult. This fact motivated us to evaluate our PPI essentiality predictions through joint analysis of PPI essentialities and drug sensitivity data. We leveraged drug sensitivity data on over 200 cell lines from the Cancer Cell Line Encyclopedia (CCLE) [22] and examined the correlations of PPI essentialities and sensitivity to 19 different compounds. We thus performed a context specific PPI analysis, removing any PPI from a cell-line specific network if either the source or target protein was deleted, had an inactivating mutation, or was not expressed at the RNA level. Because of our context-specific approach that uses genetic alterations and gene-regulation to alter PPI network topology for each cell line, the specific number of PPIs varied from cell line to cell line, but included on average 104 cell lines per PPI. We then computed the statistical significance of correlations of PPI essentiality to drug sensitivity (as defined by AUC in CCLE analysis). Interestingly, the most significant correlation (p = 4.63E-07) was that of the BRAF-KRAS interaction to PLX4720, a compound that targets BRAF. The highest absolute correlation (r = 0.71, p = 0.0013) was between the PRKDC-TP53 PPI and sensitivity to AZD6244, an inhibitor of MAPK1 (Fig 3). This strong correlation is supported by multiple studies that indicate that MAPK signaling is critical for cell cycle arrest in response to DNA damage [51, 52] and that inhibition of p38-MAPK sensitizes lung cancer cells to DNA damaging agents [53].

Comprehensive analysis of drug sensitivity correlations was performed by comparing the strength of correlations for two sets of drug/PPI pairs: 1) drug/PPIs pairs where the PPI involves a protein targeted by that drug and 2) PPIs involving no known target protein. Examining 19 CCLE compounds for which PPI essentiality data was available, we observed that target PPIs were significantly more essential than non-target PPIs by Wilcoxon Rank Sum analysis for 14 of the 19 compounds (Table 1). Random permutation analysis was performed for each set of target vs non-target protein sets to compute false discovery rates and correct for multiple hypothesis testing. Correlations of PPI essentialities for each of the 14 significant drug/PPI pairs were significant by FDR < 0.05 (Table 1).

**Table 1:** Correlations of PPI essentiality with drug sensitivities for 19 CCLE compounds with PPI essentiality data for their respective targets.

Compound	Targets	WilcoxonRankSum p-val	FDR
PF2341066	MET;ALK	9.86E-18	<0.001
Lapatinib	EGFR;ERBB2	2.16E-17	<0.001
Erlotinib	EGFR;ERBB2	3.60E-12	<0.001
ZD-6474	VEGFR;EGFR	2.19E-11	<0.001
L-685458	APH1A;NCSTN;PSEN1;PSENEN	2.90E-11	<0.001
Sorafenib	BRAF;FLT3;KDR;RAF1	3.47E-11	<0.001
PLX4720	BRAF	7.05E-07	<0.001
PD-0332991	CDK4;CDK6	1.01E-05	<0.001
AZD6244	MEK	7.65E-04	0.002
PD-0325901	MEK	4.54E-04	0.003
Nutlin-3	MDM2	9.59E-03	0.006
RAF265	BRAF;KDR	8.53E-03	0.007
LBW242	XIAP	1.46E-02	0.009
17-AAG	HSP90	3.20E-02	0.030
AEW541	IGF1R	n.s.	0.161
AZD0530	SRC;YES;FYN;LYN;BLK,FGR;LCK	n.s.	0.234
Nilotinib	ABL1;BCR;KIT	n.s.	0.286
TKI258	FLT3;FGFR1/3;VEGFR1-4	n.s.	0.540
PHA-665752	MET	n.s.	0.718

Drug sensitivities were derived from CCLE AUC data. Significance of enrichment for drug target PPI essentialities vs. non-target PPI essentialities was computed by the Wilcoxon Rank Sum test, and was significant for 14 of 19 compounds. Target and non-target gene sets were randomly permuted 1000 times to compute FDR and correct for multiple hypothesis testing.

### Genetic alterations

**Fig 4:**
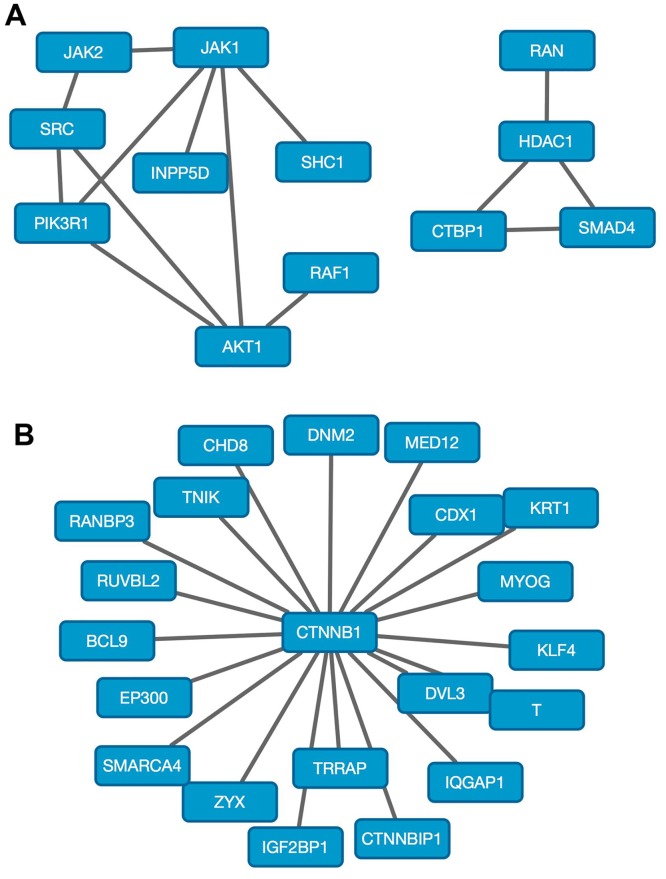
PPI networks associated with genetic mutations. (A) Networks of PPIs most increased in essentiality in cells with mutation or loss of the PTEN tumor suppressor gene. The 14 most significant PPIs are shown. Significant differences in PPI essentiality were computed in GenePattern [54]. Networks were visualized with Cytoscape [55]. (B) Networks of PPIs most increased in essentiality in cell lines with mutation or loss of the APC tumor suppressor gene. The 20 most significant PPIs are shown.

To further verify MEDICI essentiality predictions, we investigated the associations between known genetic alterations and PPI essentialities. We divided the PPI essentiality data from the 165 cell lines analyzed in Fig 2 based on the presence or absence of wild type PTEN or APC tumor suppressor genes. We hypothesized that cell lines with mutant PTEN would have increased dependence on PPIs in the PI3K-AKT pathway, while those with mutant APC would exhibit increased dependence on β-catenin (CTNNB1). Indeed, when we analyzed PPI essentiality data for cell lines with PTEN mutations (n = 25) relative to those with wild-type PTEN (n = 140), we observed that the most essential PPIs were related to PI3K-AKT signaling (Fig 4A). Top PPIs included JAK1-AKT, JAK1-PIK3R1, SRC-AKT1, and PIK3R1-AKT1 (FDR(BH) = 2.13e-4)). Examining cell lines with mutant APC (n = 14) compared to those with wild-type APC (n = 151), the 95 most significant PPIs all involved interactions with β-catenin (FDR(BH) = 2.13e-4)). The top 20 PPIs from this analysis are shown in Fig 4B.

### Clinical outcome analysis

**Fig 5:**
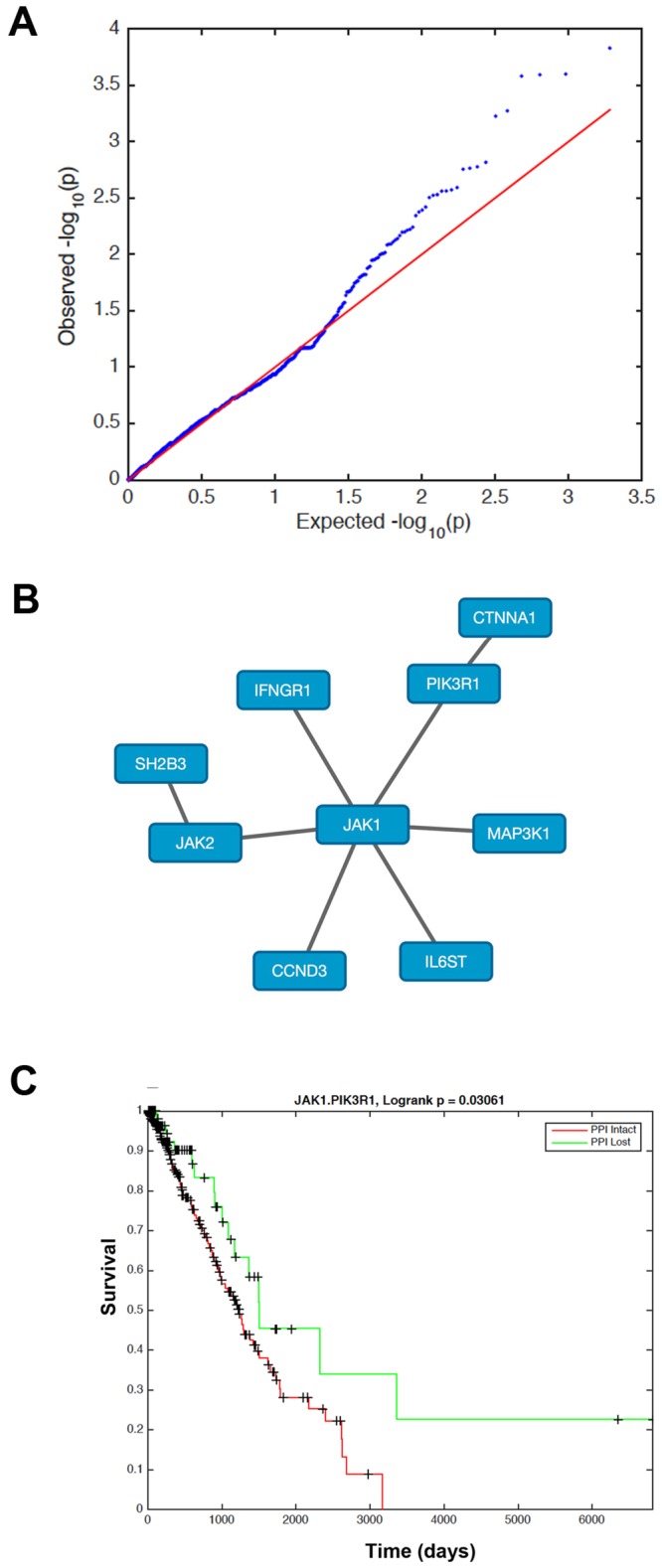
PPI Essentiality association with patient survival. (A) QQ plot of observed vs. expected log-rank p-values for LUAD patients split based on 5798 PPIs. (B) Network of PPIs with significant log-rank p-value for discriminating survival for TCGA LUAD patients that is centered on JAK1. (C) Kaplan-Meier curve of TCGA LUAD patients separated based on the presence or absence of the JAK1-PIK3R1 PPI. Patients without the JAK1-PIK3R1 PPI have improved survival compared to patients who retain this PPI.

We next wanted to address whether loss of a particular PPI might confer a survival advantage to lung cancer patients. To test whether this was the case, we analyzed genomic data from the lung adenocarcinoma (LUAD) [56] datasets from The Cancer Genome Atlas (TCGA) Network. We separated patients into those with or without each of the PPIs in our superpathway network based on the presence or absence of nonsense mutations, frameshift mutations, or homozygous deletions for each of the partner proteins. We then computed log rank p-values for survival based on whether each of the PPIs was present or absent. QQ plots of observed vs expected log-rank p-values for LUAD patients (Fig 5A) indicate a strong trend of more significant p-values than those expected by chance. However, log rank p-values corrected for multiple tests using Benjamini-Yekutieli correction [57] did not achieve statistical significance, and should therefore be interepreted with caution. We then examined the set of PPIs with nominal log rank p-value < 0.05 that were also essential in lung adenocarcinoma cell lines based on our analysis of Achilles data. For LUAD patients, 15 PPIs were met this criterion (S2 Table, Fig 5B). Of those 15 PPIs, six included JAK1 as a binding partner, two included JAK2, and one was the JAK1-JAK2 interaction. It has recently been shown that inhibition of JAKs attenuates growth of small cell lung cancers *in vitro* and *in vivo* [58], and that activation of JAK signaling induces resistance to EGFR mutations in non-small cell lung cancers [59]. Also of potential interest is an ATM-NBN interaction that is involved in double-strand DNA break repair and PI3KR1 interactions involved in activation of the PI3K pathway.

## Discussion

While most cancer drugs either cause DNA damage or target the active sites of enzymes such as protein kinases, a growing number of new compounds interfere with critical protein-protein interactions (PPIs). Although the pharmaceutical industry has been reluctant to develop compounds that target PPIs, it is becoming clear that these types of drugs can provide lethal and specific targeting of cancer cells. Examples of these compounds include Navitoclax (ABT-263) [60], which inhibits BCL2-BAX interactions; Nutlin-3 [61], which blocks MDM2-TP53 interactions; PRI-724 [62], which interferes with β-catenin-CBP interactions; and JQ-1 [13] and I-BET726 [63], which prevent BRD4 binding to acetylated histones. These new compounds are showing promise for treatment of previously difficult to target pathways, including the p53 tumor suppressor [12], Wnt pathway [62], and Myc oncogene [64].

Nevertheless, while there may be on average approximately 10,000–15,000 proteins expressed in any given cell type, there are likely an order of magnitude more PPIs, or over 600,000 PPIs in mammalian cells [65]. Thus, prioritizing which PPIs may be important for drug development is an even greater challenge than identifying individual proteins as potential drug targets. The network topology of cancer drug targets has been shown to be more hub-like than non-drug targets, suggesting that PPIs can be useful in identification of drug target proteins for cancer [66] and neurological diseases [67]. Thus, combinations of computational and experimental methods will be critical to enable identification of the most promising PPIs that are essential to the survival of cancer cells.

In this study we have described a novel approach to computationally infer the essentiality of PPIs based on a combination of network topology and high-throughput screening gene knockout studies. This study represents our first attempt to provide a framework to predict and prioritize PPIs as potential targets for therapeutic discovery, which is expected to mature with the incorporation of additional datasets and biological insights. While this approach is quite general, here we have applied it to only a single large dataset from Project Achilles [8], which by definition, when integrated with our superpathway, limited our analyses to include only 7906 PPIs. Future studies integrating additional high throughput datasets from siRNA, shRNA, and/or CRISPR/Cas9 screens would integrate multiple complementary datasets that may provide more robust PPI essentiality predictions. Another limitation of our current study is that shRNA screens can have off-target effects. For this reason, we limited our analysis to only those Achilles shRNA targets that were validated by multiple shRNAs that targeted the same genes.

Our analysis is further limited by our understanding and knowledge of the complete PPI network within cancer cells. A variety of approaches including mass spectrometry, yeast two-hybrid, and TR-FRET methodologies are continually expanding and improving our understanding of PPI networks. As our grasp on the topology of these PPI networks improves, the potential accuracy, breadth, and utility of inferences regarding PPI essentiality using our methods described here will naturally improve as a result.

Here, we have developed cell-line specific network topologies by removing PPIs in which one partner gene is deleted, mutated, or not expressed. However, other than this, we do not account for differences in gene expression or abundance of various PPI partner proteins. Additional ways to potentially refine our approach would involve analysis of the transcriptional profile of cell lines to deduce which subset of PPIs that are possible within a cell are actually taking place. Currently, our approach assumes that two proteins will interact within a cell line if they are known to interact, are expressed and are not mutated. However, signal transduction pathways can greatly modify the constellation of potential PPIs within a cell, and thus layering pathway activation data onto our current network topologies could improve our approach.

Even so, the analyses presented here indicate that the sets of PPI essentialities that we have computed have strong biological relevance. The correlations of essentialities to drug sensitivities and genetic mutations validate our overall approach and provide new insights into potentially important PPIs that could be used for guiding cancer drug target development.

## Supporting Information

S1 Fig Unsupervised hierarchical clustering of all non-zero 5798 PPIs across 165 cell lines. PPI essentiality data was median centered and clustered by average correlation. Red indicates higher essentiality and blue indicates lower essentiality.
(TIF)

S2 Fig Unsupervised hierarchical clustering of 5711 genes across 165 cell lines based on Achilles direct shRNA sensitivity data. Cell lines cluster primarily by tissue of origin. The main strong gene clusters were driven by sets of transcription factors, ribosomal proteins, or genes of unknown function.
(TIF)

S3 Fig Unsupervised hierarchical clustering of the 343 proteins with the top average shRNA sensitivity across all cell lines (<-0.05). Cell lines cluster primarily by tissue of origin. Few distinct gene-based clusters are present, with the exception of one small cluster containing MHC class I receptors.
(TIF)

S4 Fig Comparison of protein and protein-interaction essentialities. To evaluate the utility of MEDICI, we compared estimated PPI essentiality values to the experimentally measured essentialities for individual proteins. Each point in the scatter plots of panels (A-D) represents the essentiality rank of a single PPI versus and the max protein essentiality rank of the constituent proteins in that interaction. Color indicates density of points. Plots for four cell lines (A) A549 lung cancer, (B) BXPC3 pancreatic cancer, (C) MKN7 stomach cancer, and (D) U87MG glioma cell lines are shown. The EGFR-ERBB3 PPI is indicated in each plot with an arrow. A large number of PPIs appear in the upper-left of each panel in which PPI essentiality significantly exceeds max constituent protein essentiality. These entries provide insights into interaction-specific sensitivity that cannot be readily observed in the measure protein essentiality data. Ranks/percentiles were used to generate these plots to avoid any consistent biases in the magnitude of PPI versus protein essentialities.
(TIF)

S1 Table Essentiality values for 7906 PPIs across 206 cancer cell lines. NaN indicates that one of the proteins in the interaction is deleted, mutated, or not expressed in that cell line.
(XLS)

S2 Table PPIs with nominally significant log-rank p-values for LUAD patient survival PPI and KS. (XLSX)

S3 Table PPI-MPER are provided for 7906 PPIs and 206 cell lines. Also shown are PPI rank percentiles and maximum protein essentiality rank percentiles.
(TXT)

S1 Methods Detailed supplementary methods and convergence proof. (DOCX)
