# Supplementary figures and images for: MEDICI: Mining Essentiality Data to Identify Critical Interactions for Cancer Drug Target Discovery and Development
Source: PLoS One. 2017 Jan 24;12(1):e0170339. doi: 10.1371/journal.pone.0170339 (PMC5261804; doi:10.1371/journal.pone.0170339)

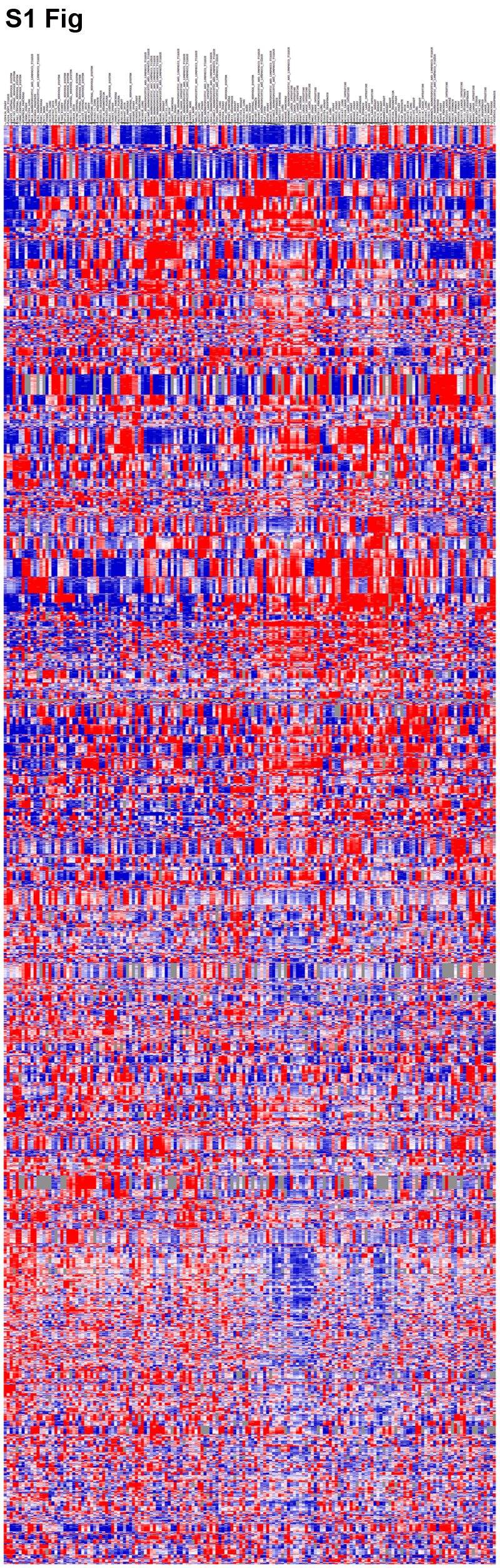

Supplement: S1 Fig — PPI essentiality data was median centered and clustered by average correlation. Red indicates higher essentiality and blue indicates lower essentiality. (TIF) [file pone.0170339.s001.tif]

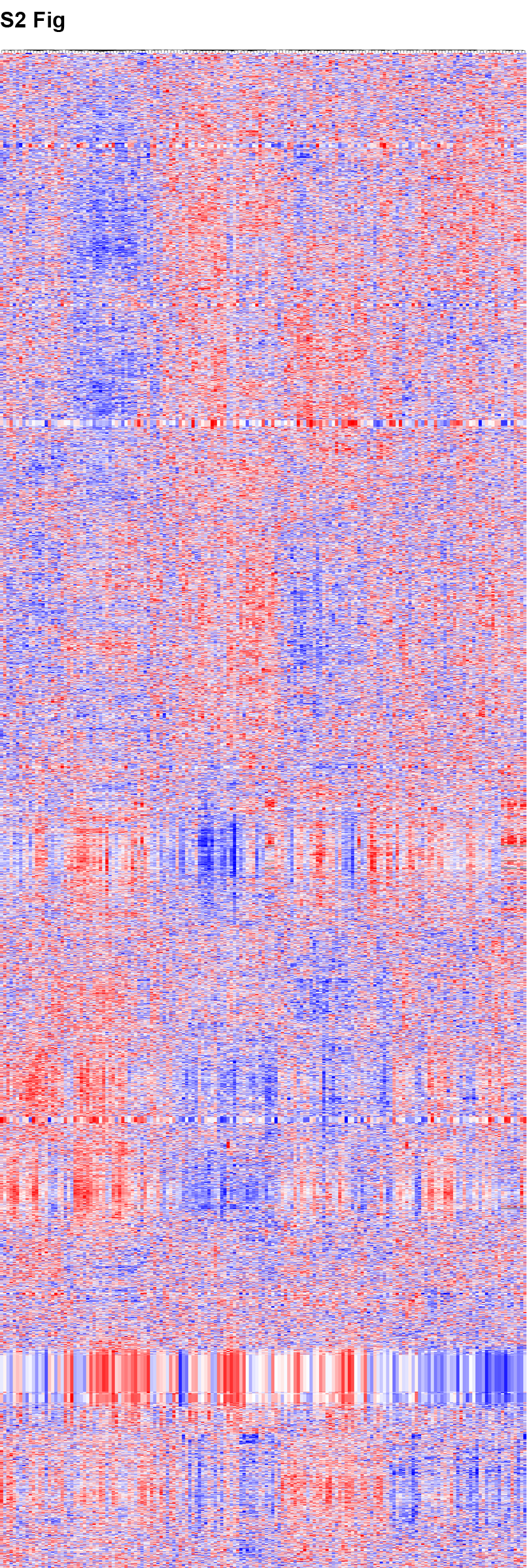

Supplement: S2 Fig — Cell lines cluster primarily by tissue of origin. The main strong gene clusters were driven by sets of transcription factors, ribosomal proteins, or genes of unknown function. (TIF) [file pone.0170339.s002.tif]

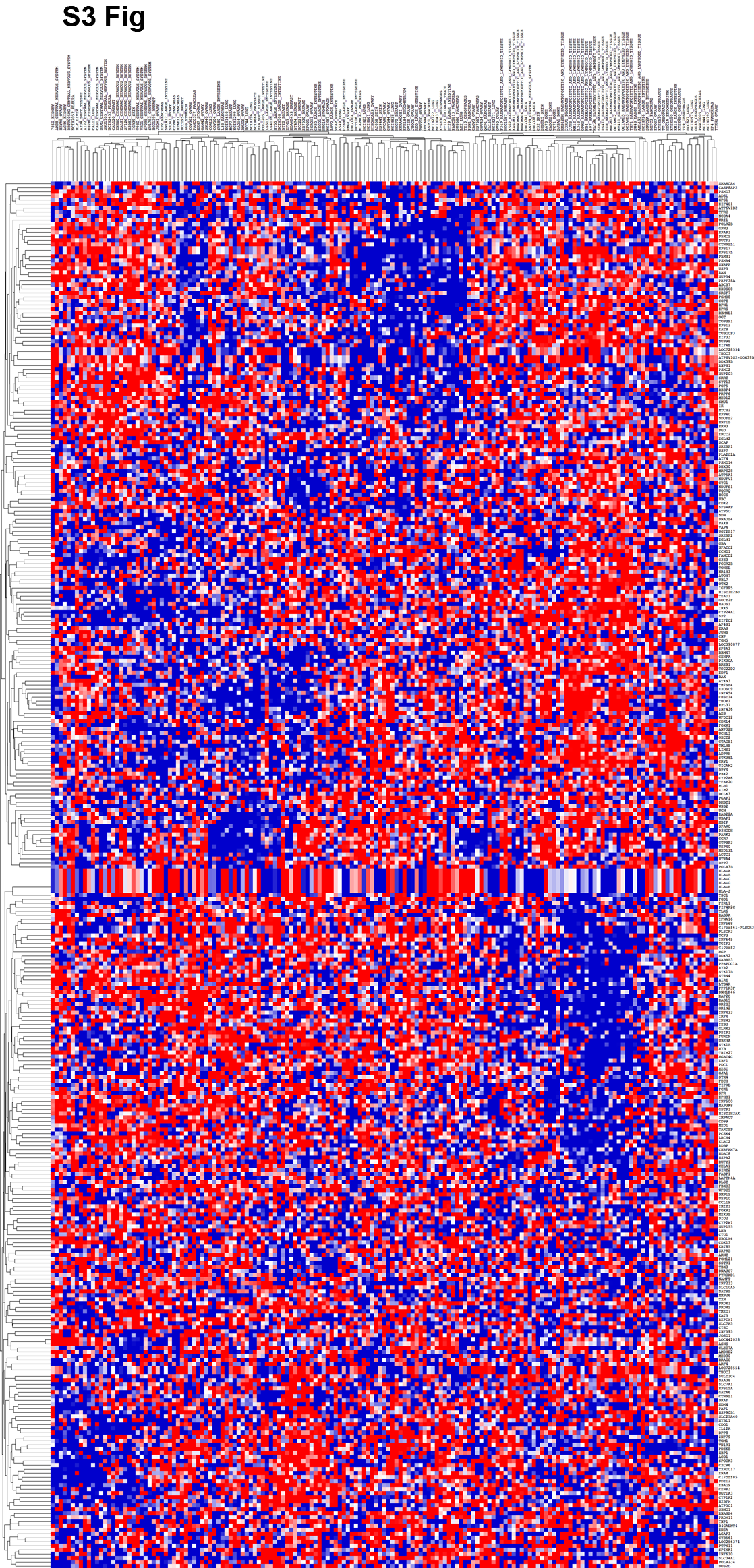

Supplement: S3 Fig — Cell lines cluster primarily by tissue of origin. Few distinct gene-based clusters are present, with the exception of one small cluster containing MHC class I receptors. (TIF) [file pone.0170339.s003.tif]

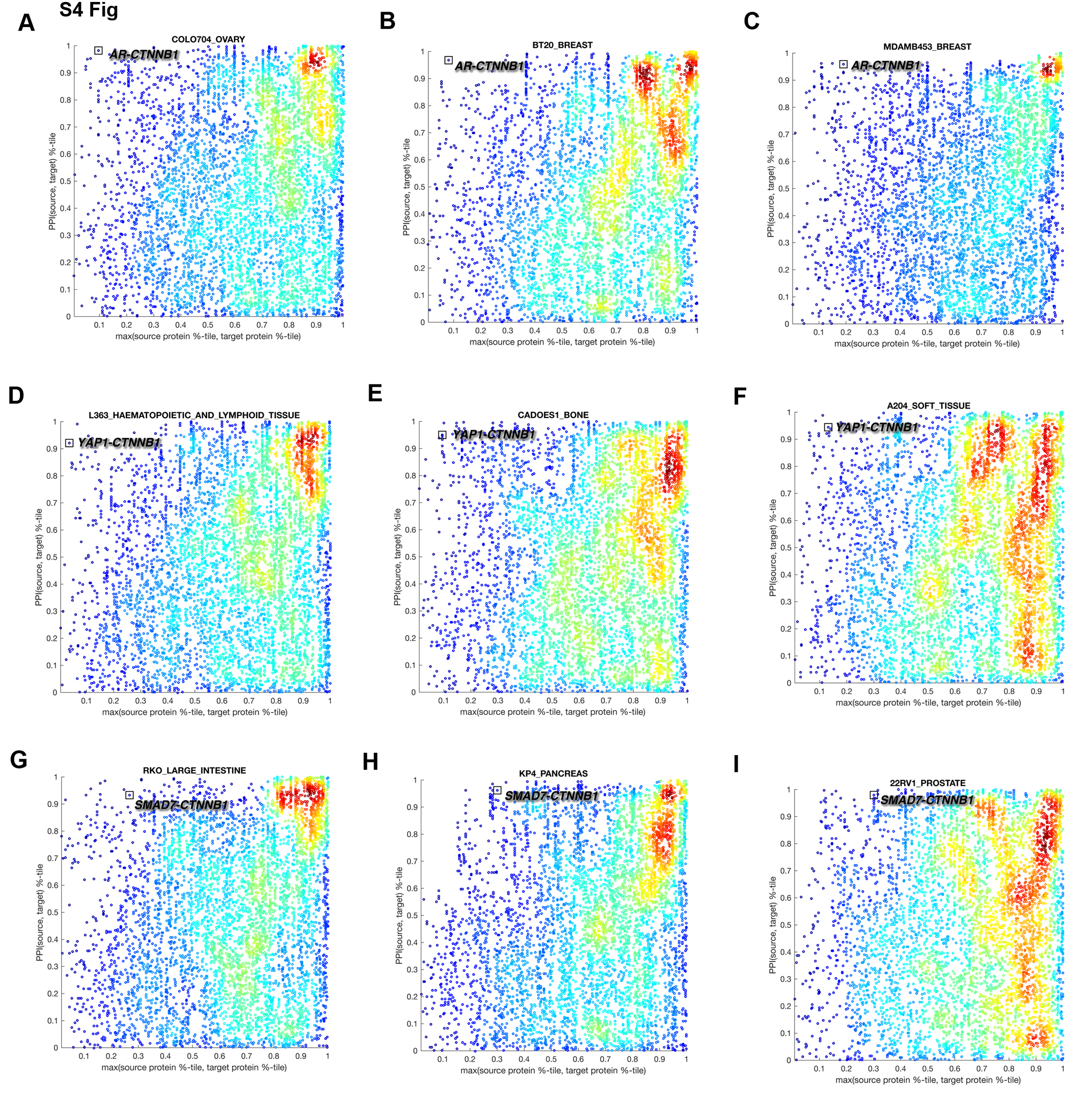

Supplement: S4 Fig — To evaluate the utility of MEDICI, we compared estimated PPI essentiality values to the experimentally measured essentialities for individual proteins. Each point in the scatter plots of panels (A-D) represents the essentiality rank of a single PPI versus and the max protein essentiality rank of the constituent proteins in that interaction. Color indicates density of points. Plots for four cell lines (A) A549 lung cancer, (B) BXPC3 pancreatic cancer, (C) MKN7 stomach cancer, and (D) U87MG glioma cell lines are shown. The EGFR-ERBB3 PPI is indicated in each plot with an arrow. A large number of PPIs appear in the upper-left of each panel in which PPI essentiality significantly exceeds max constituent protein essentiality. These entries provide insights into interaction-specific sensitivity that cannot be readily observed in the measure protein essentiality data. Ranks/percentiles were used to generate these plots to avoid any consistent biases in the magnitude of PPI versus protein essentialities. (TIF) [file pone.0170339.s004.tif]
