# Supplementary material for: MEDICI: Mining Essentiality Data to Identify Critical Interactions for Cancer Drug Target Discovery and Development
Source: PLoS One. 2017 Jan 24;12(1):e0170339. doi: 10.1371/journal.pone.0170339 (PMC5261804; doi:10.1371/journal.pone.0170339)
Supplement: S1 Methods — (DOCX) [file pone.0170339.s008.docx]

**S1 Methods**

### Convergence Proof Outline

Suppose the dual graph of our original network has $m$nodes. We define vector $E = (e_{1},e_{2},e_{3},..., e_{m})$be the score of nodes. The edge weight between nodes $i$ and $j$ are denoted as $w_{ij}$. Define the weight matrix $W={[w_{ij}]}_{\left( i,j \right)\in\left\{ 1,..,m \right\}\times\{1,..,m\}}$and the diagonal matrix $D$ where $D_{ii}=\sum_{j\neq i}^{m} w_{ij}$. The normalized matrix $P=WD^{-1}$is a stochastic matrix (columns are summed to 1). Then the update rule can be written in matrix form:

$$e^{(t+1)}=(1-\alpha)e^{(t)}+\alpha e^{(t)}P$$

$$e^{(t+1)}=((1-\alpha) I+\alpha P)e^{(t)}$$

$$Q=(1-\alpha) I+\alpha P$$

$$e^{(t+1)}=Qe^{(t)}$$

$I$is the $m\times m$identity matrix. Since both $P$and $I$are stochastic$Q$is also a stochastic matrix with eigenvalues less than or equal to 1 the updating process will converge. At the convergence point $e^{*}$, we should have $e^{*}=Qe^{*}$. Therefore $e^{*}$ is the eigenvector of $Q$ corresponding to the eigenvalue $\lambda=1 (i.e. the largest eigenvalue)$.

*Statistical tests*

To evaluate these PPIs in patient samples, we used mutational, CNV and mRNA expression profiles to infer which PPIs are absent in each patient. A Cox proportional hazards analysis was then used to measure the prognostic significance of these groups. PPIs from lung adenocarcinoma derived lines were ranked to identify the most essential PPIs, KS statistics were computed for ranks across these lines, and a positive KS statistic > 0.5 was used as a threshold for significant PPIs in LUAD cell lines.

### 
